# Validation of the NAT Chagas IVD Kit for the Detection and Quantification of *Trypanosoma cruzi* in Blood Samples of Patients with Chagas Disease

**DOI:** 10.3390/life13061236

**Published:** 2023-05-24

**Authors:** Otacilio C. Moreira, Alice Gomes Fernandes, Natalia Lins da Silva Gomes, Carolina Messias dos Santos, Thiago Jacomasso, Alexandre Dias Tavares Costa, Lucas de O. Rossetti Nascimento, Alejandro Marcel Hasslocher-Moreno, Pedro Emmanuel Alvarenga Americano do Brasil, Luis Gustavo Morello, Fabricio Klerynton Marchini, Marco Aurelio Krieger, Constança Britto

**Affiliations:** 1 Real Time PCR Platform RPT09A, Laboratory of Molecular Virology and Parasitology, Oswaldo Cruz Institute/Fiocruz, Rio de Janeiro 21040-900, Brazil; 2 Laboratory of Molecular Biology and Endemic Diseases, Oswaldo Cruz Institute/Fiocruz, Rio de Janeiro 21040-900, Brazil; 3 Instituto de Biologia Molecular do Paraná, Curitiba 81350-010, Brazil; 4 Laboratory for Applied Science and Technology in Health, Carlos Chagas Institute/Fiocruz, Curitiba 81310-020, Brazil; 5 Laboratory of Clinical Research in Chagas Disease, Evandro Chagas National Institute of Infectious Diseases/Fiocruz, Rio de Janeiro 21040-360, Brazil

**Keywords:** Chagas disease, molecular diagnostics, NAT Chagas, real-time PCR, parasite load

## Abstract

In the absence of validated biomarkers to control the cure of Chagas disease, PCR-based diagnosis is being used as the main tool for an early indication of therapeutic failure. However, since it is considered a technique of complex reproducibility, mainly due to difficulties in establishing accurate controls to guarantee the quality of the reaction, the use of PCR for Chagas disease diagnosis is restricted to specialized centers. In an effort to disseminate the molecular diagnosis of Chagas disease and its applications, new diagnostic kits based on qPCR have been made available in the market in recent years. Here, we show the results of the validation of the NAT Chagas kit (Nucleic Acid Test for Chagas Disease) for the detection and quantification of *T. cruzi* in blood samples of patients suspected of Chagas disease infection. The kit, composed of a TaqMan duplex reaction targeting the *T. cruzi* satellite nuclear DNA and an exogenous internal amplification control, presented a reportable range from 10^4^ to 0.5 parasite equivalents/mL and a limit of detection (LOD) of 0.16 parasite equivalents/mL of blood. In addition, the NAT Chagas kit detected *T. cruzi* belonging to all six discrete typing units (DTUs—TcI to TcVI), similarly to the in-house real-time PCR performed with commercial reagents, which has been selected as the best performance assay in the international consensus for the validation of qPCR for Chagas disease. In the clinical validation presented here, the kit showed 100% sensitivity and 100% specificity when compared to the consensus in-house real-time PCR assay. Thus, the NAT Chagas kit, which is produced entirely in Brazil under the international standards of good manufacturing practices (GMP), appears as an excellent alternative to enable the molecular diagnosis of Chagas disease in public and private diagnostic centers, as well as to facilitate the monitoring of patients under etiological treatment participating in clinical trials.

## 1. Introduction

Chagas disease (CD), also known as American trypanosomiasis, is an infectious disease caused by the protozoan parasite *Trypanosoma cruzi* that affects an estimated 6–7 million people and puts another 75 million at risk of infection [1]. CD is endemic in Latin American rural areas, with Argentina, Brazil, Mexico, and Bolivia having the highest prevalence of infected people [2]. However, as globalization and migration mobility have accelerated, nowadays, CD has spread to countries where it was not previously endemic, such as the United States, Spain, Japan, and Australia [2,3,4,5]. The cardiac form of the disease, known as chronic Chagas cardiomyopathy (CCC), is the most common and severe form of CD and is one of the leading causes of morbidity and mortality in endemic areas [3].

The diagnosis of CD depends on the stage of the disease. In the acute phase, where parasitemia is high, direct parasitological methods are recommended for the detection of trypomastigotes [6,7]. In the chronic phase, characterized by a reduction in parasitemia and the intermittent release of trypomastigote forms into the bloodstream [8], the diagnosis is essentially performed by serological tests based on IgG detection. Conventional serological techniques, such as indirect immunofluorescence (IIF), indirect hemagglutination (IHA), and enzyme-linked immunosorbent assays (ELISA), present suboptimal specificity. Thus, the current WHO/PAHO recommendation is the simultaneous use of at least two serological tests using different technical principles to define the diagnosis during the chronic phase of CD [9]. 

Since the 1990s, PCR has been used as a molecular tool for CD diagnosis due to its high sensitivity and specificity for the detection of *T. cruzi* DNA and its potential application in the monitoring of trypanocidal chemotherapy [10,11,12]. PCR is a rapid detection method and has become frequently used in the molecular diagnosis of CD over the last few years [13,14]. Due to its greater sensitivity and speed in generating results, compared to classical parasitological methods, PCR-based assays have been shown to be useful in different scenarios of *T. cruzi* infection, for instance, to early diagnose congenital transmission in newborns [15,16,17]; in the diagnosis of oral infection [18,19]; for the screening of *T. cruzi*-contaminated food such as açai, sugar cane, and bacaba [20,21]; for prior detection of acute infection in recipients of transplanted organs from infected donors [22,23]; for monitoring reactivation in chronic immunosuppressed patients due to organ transplant or *T. cruzi*/HIV coinfection [24,25]; and for the assessment of response to treatment, since serological conversion (negative) in treated patients with chronic CD can take decades to occur [3,26,27]. 

During the chronic phase, PCR generates positive results in 40 to 70% of patients previously diagnosed by conventional serology. The variation in PCR positivity is dependent on the degree of parasitemia, sample volume, DNA purification method, target region to be amplified, the characteristics of the study populations, and the high genetic variability observed between the discrete typing units (DTUs) of the parasite [13,15,26,28,29]. In the case of inconclusive results of serological tests, the Brazilian Guideline for the Diagnosis of Chronic Chagas Disease determines that the PCR methodology can be used to confirm the results [6]. 

Quantitative real-time PCR (qPCR) was developed to enable the detection and quantification of parasite DNA from clinical samples, using intercalating dyes or labeled probes, in the presence of standard curves with known concentrations of the parasite [30,31,32,33]. This methodology, like conventional PCR, has varying levels of analytical specificity and sensitivity [30,31,34]. Therefore, its application in clinical routine requires previous analytical and clinical validation studies [24,35]. In CD, after standardization and analytical validation, most qPCR protocols are directed to the amplification of nuclear satellite DNA (SatDNA) or kinetoplast minicircle DNA (kDNA) sequences of *T. cruzi*—including an exogenous internal amplification control (IAC). Consequently, satDNA and kDNA-based protocols have been frequently used, particularly for the monitoring of trypanocidal treatment [32,33,36]. Target Product Profiles (TPPs) for the molecular diagnosis of CD have been proposed for acute and congenital cases, for the chronic phase, and to monitor response to trypanocidal treatment [37,38]. These TPPs consider “minimum” and “optimal” needs related to the epidemiological characteristics of patients and clinical groups, sensitivity and specificity of the diagnostic assay, sample volume and clinical specimens, storage conditions, transport and storage, infrastructure, the degree of technical skill of operators, and, finally, the need to report qualitative or quantitative results and for parasite genotyping [26].

In 2011, an international workshop organized by PAHO/WHO was held in Buenos Aires, with the participation of 26 laboratories from 14 countries with previous experience in PCR techniques. The aim of this meeting was to evaluate the performance of qPCR strategies in duplex format, based on the use of TaqMan probes for the detection and quantification of parasite load in blood samples from patients with acute and chronic Chagas disease [36]. From this initiative, a TaqMan assay targeting both the *T. cruzi* satDNA and IAC was recommended by specialists as the most accurate assay for the molecular diagnosis of CD in all endemic countries. Based on this consensus methodology, the Nucleic Acid Test for Chagas disease (NAT Chagas) kit was developed as a TaqMan duplex reaction targeting the *T. cruzi* satellite nuclear DNA (satDNA) and an IAC. The kit was entirely produced in Brazil within the international standards of good manufacturing practices and was developed for the in vitro diagnosis (IVD) of CD from the use of guanidine-EDTA blood (GEB) samples. 

This study presents the main experiments performed for the validation of the NAT Chagas kit, using reference *T. cruzi* strains from six DTUs (TcI to TcVI) and GEB samples of chronic CD patients from different states of Brazil, infected with genetic *T. cruzi* lineages belonging to different DTUs. Based on the successful history of the NAT kits family, the NAT Chagas kit represents a milestone for endemic countries or places with imported cases of CD, since its production and distribution have great potential to facilitate the molecular diagnosis and follow-up of patients undergoing treatment.

## 2. Materials and Methods

Ethical statements. The study in which the clinical samples were collected was approved by the ethical committee of the Fundação Oswaldo Cruz (CEP 007/2007), according to the principles expressed in the Declaration of Helsinki. Written informed consent forms were signed by all the study subjects. All samples were pre-existent at the time of the present study and were anonymized before being processed. 

Study design. The performance of NAT Chagas prototype kit was evaluated in parallel with the in-house qPCR assay, recommended by the international workshop organized by PAHO/WHO [36]. The analytical validation was performed using *T. cruzi* epimastigotes from the six DTUs (TcI to TcVI). For the specificity analysis, the following pathogens were also used *Leishmania braziliensis*, *Leishmania amazonensis*, *Leishmania guyanensis*, *Leishmania infantum*, *Cyclospora cayetanensis*, *Eimeria acervuline*, *Eimeria tenella*, *Plasmodium falciparum*, *Toxoplasma gondii*, and *Plasmodium vivax*. For the clinical validation, GEB samples from 50 subjects were used, comprising 32 individuals with positive and 18 individuals with negative results for CD, with two independent commercial serological tests (indirect immunofluorescence: Imuno-con (WAMA Diagnóstica, São Paulo, Brazil) and enzyme-linked immunoassay (ELISA): Chagastest Recombinant ELISA (Wierner Lab., Rosario, Argentina)), according to the manufacturer’s recommendations. Patients with CD were from different regions of Brazil (Northeast, Southeast, Midwest and South) and infected with different *T. cruzi* DTUs (TcII, TcV, TcVI, TcII + TcVI), presenting the indeterminate or chronic cardiac forms of Chagas disease. Individuals with negative serology were from a non-endemic area, in the state of Rio de Janeiro. All qPCRs were run using the same number of samples and in two technical replicates. The same DNA sample, extracted from *T. cruzi* epimastigotes or GEB specimens, was evaluated in parallel for both the qPCR assays (NAT Chagas and in-house qPCR).

*Trypanosoma cruzi* cultivation. Epimastigote forms of *T. cruzi* strains/clones (Dm28c, TcI; Y, TcII; INPA 3663, TcIII; INPA 4167, TcIV; LL014, TcV; and CL-Brener, TcVI) were cultivated in liver infusion tryptose medium supplemented with 10% inactivated bovine fetal serum for 5 days at 28 °C. Parasites were then pelleted, washed three times in phosphate-buffered saline, and counted in a Neubauer chamber before use.

DNA extraction. Ten milliliters of peripheral blood samples was harvested in EDTA-K2 tubes and immediately mixed with an equal volume of 6 M Guanidinium Hydrochloride/0.2 M EDTA, pH 8.0 (GEB) provided in the NAT Chagas kit. After 48 to 72 h at room temperature, GEB samples were boiled for 15 min and stored at 4 °C for DNA extraction and PCR analysis. Prior to DNA extraction from 300 µL of GEB samples, an aliquot of 5 µL of IAC provided in the NAT Chagas kit (2 × 10^6^ copies/μL—Table 1) was added, and total DNA was extracted using the High Pure PCR Template Preparation kit (Roche Diagnostics Corp., Indianapolis, IN, USA), according to the manufacturer’s recommendations. In the final step, DNA was eluted in 100 µL of elution buffer and stored at −20 °C until use. 

Real-time qPCR assays. NAT Chagas: This is a ready-to-use kit that is provided with a prepared mix of reagents with all the necessary components to set up the assay. It has been developed into a commercial product manufactured by the Instituto de Biologia Molecular do Paraná (IBMP, Curitiba, Brazil) and released to the market as “NAT Chagas kit” for IVD use, after approval by the “Agência Nacional de Vigilância Sanitária”, ANVISA, Brasília-DF, Brazil, under the Registration number 80780040010. For the NAT Chagas, a TaqMan-based duplex qPCR assay targeting *T. cruzi* satDNA sequence (FAM/NFQ-MGB) and IAC (HEX/NFQ-MGB) was developed and standardized. The reaction mixture was prepared as recommended by the manufacturer: 4 µL nuclease-free water (from the kit), 1 µL Oligomix Chagas [20X], and 10 µL Mix NAT Chagas [2X] were mixed with 5 μL DNA, for a final volume of 20 μL. Cycling conditions were as follows: a first step at 95 °C for 10 min, followed by 45 cycles at 95 °C for 15 s and at 60 °C for 1 min. The amplifications were carried out in an ABI Prism 7500 Fast device (Applied Biosystems, Waltham, MA, USA). The threshold was set at 0.2 for *T. cruzi* and 0.02 for IAC targets, respectively.

In-house assay: For the reference in-house qPCR assay [36], 5 μL DNA was mixed with 10 μL Fast Start Universal Probe Master Mix (Rox) (2X) (Roche, Basel, Switzerland), 300 nM Cruzi1 (5′-AST CGG CTG ATC GTT TTC GA-3′), and Cruzi2 (5′-AAT TCC TCC AAG CAG CGG ATA-3′) primers and 100 nM Cruzi3 probe (5′-FAM CAC ACA CTG GAC ACC AA-NFQ-MGB-3′), targeting the *T. cruzi* satDNA, 100 nM IAC Fw (5′-ACC GTC ATG GAA CAG CAC GTA-3′), 100 nM IAC Rv (5′-CTC CCG CAA CAA ACC CTA TAA AT-3′) and 50 nM IAC Tq (5′-VIC-AGC ATC TGT TCT TGA AGG T-NFQ-MGB-3′), targeting the IAC, for a final volume of 20 μL. Cycling conditions were as follows: a first step at 95 °C for 5 min, followed by 40 cycles at 94 °C for 15 s and at 58 °C for 1 min. The amplifications were carried out in an ABI Prism 7500 Fast device (Applied Biosystems, USA). The threshold was set at 0.02 for both targets.

Controls and qPCR analysis. For DNA extraction, one negative extraction control (seronegative blood for CD) was included in each DNA extraction batch, together with 11 GEB samples. 

For the NAT Chagas kit, two positive controls (synthetic double-stranded *T. cruzi* satDNA conserved sequence, at 100 and 10 copies/μL), supplied with the kit, were used in each assay. To obtain the 166-bp sequence for the synthetic DNA controls, the satDNA repeats sequences from strains and clones representing *T. cruzi* DTUs I to VI available at the GenBank were aligned, showing the high conservation of the repeats [39]. As an exogenous control, a 181-bp double-stranded synthetic DNA sequence, also supplied with the kit, was used, as described in the DNA extraction section. The IAC was derived from a sequence of *Arabidopsis thaliana* aquaporin, as previously reported [32]. Table 1 shows the sequences of both controls, including the ID and description of the reference sequences from Genbank. 

For the in-house assay, two positive controls (DNA extracted from *T. cruzi* epimastigotes—Y strain, at 10 and 1 femtograms/μL) were used in each assay. In addition, the same synthetic IAC double-stranded DNA was used, as described above. In both qPCR assays, Negative Template Controls (NTC) were added, using 5 μL nuclease-free water instead the DNA sample, and the reactions were performed in technical duplicates for each DNA sample and controls.

In both assays, a sample was considered valid when the IAC target was amplified as expected (between Cts 17.8 and 24.3 for the NAT Chagas and Cts 20 and 22 for the in-house assay). A valid sample was considered positive (detectable *T. cruzi* DNA) when the amplification curve for the *T. cruzi* target exceeded the fluorescence threshold, resulting in a Ct value.

Standard curves for the qPCR assays. In this study, the following two different standard curves were used in triplicate. (a) Cultured parasites: the first standard curve was prepared, spiking a GEB-seronegative sample with 10^5^ parasite equivalents (Par. Eq./mL) per mL of blood (*T. cruzi* Y strain, TcII). DNA was purified as described above for clinical samples, and serial dilutions in a “DNA solution matrix” obtained from a pool of GEB-seronegative samples were performed, ranging from 10^4^ to 0.5 Par. Eq./mL. (b) Synthetic satDNA: the second standard curve was prepared from synthetic *T. cruzi* satDNA double-strand template, as reported in [39]. Briefly, DNA obtained from GEB-seronegative samples was mixed with the synthetic *T. cruzi* satDNA template at 10^8^ copies/mL, and 1/10 serial dilutions were performed as described for the standard curve generated by DNA extracted from *T. cruzi* parasites, ranging from 10^5^ to 1 satDNA copies/mL.

Statistical analysis. All experiments were performed in at least two technical replicates. To assess the normality of the data, the Shapiro–Wilk test was used. To determine whether there were any significant statistical differences between three or more groups, unpaired one-way ANOVA with Tukey’s multiple comparison with a 95% confidence interval (CI) was used, and Student’s *t*-test or Mann–Whitney Rank Sum tests were used to analyze the statistical significance of the observed differences between two groups. All statistical tests were performed using SigmaPlot 14.0 for Windows (SPSS, Chicago, IL, USA). The data were expressed as mean and standard deviation (SD), and differences were considered statistically significant when *p* < 0.05.

## 3. Results

### 3.1. Analytical Validation

**Figure 1:**
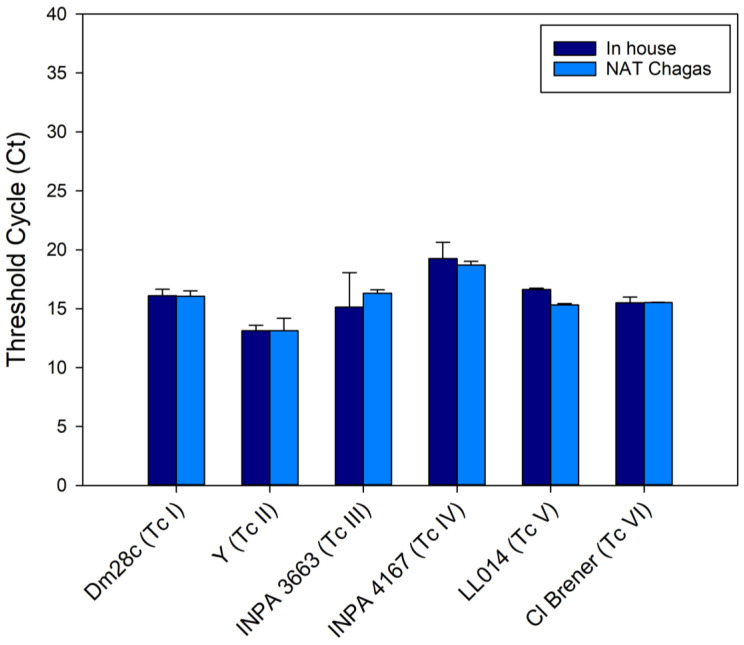
Detection of *T. cruzi* satDNA from different DTUs with both NAT Chagas and the in-house qPCR (*n* = 3). DNA samples were extracted from parasites at 10^4^ parasites/mL, belonging to strains TcI to TcVI (Dm28c, Y, INPA 3663, INPA 4167, LL014, Cl Brener strains/clones, respectively), and analyzed in parallel with NAT Chagas and the in-house assay.

Following the optimization and standardization of the best assay conditions, such as reagent concentration, cycling conditions, and the production of synthetic DNA controls, the NAT Chagas kit was validated with *T. cruzi* samples belonging to different DTUs (Figure 1). For this purpose, *T. cruzi* epimastigotes obtained from axenic culture (strains or clones Dm28c, Y, INPA 3663, INPA 4167, LL014, and CL Brener), classified as TcI to TcVI, respectively, were used. With the NAT Chagas kit, lower Ct values were observed for strain Y (TcII), 13.11 ± 1.06, and higher Ct values were observed for strain INPA-4167 (TcIV). Similarly, with the in-house assay, lower (Ct 13.12 ± 0.46) and higher (19.26 ± 1.38) Ct values were achieved for the same parasite strains. For all *T. cruzi* DTUs, no significant difference was observed in the amplifications (Ct values) between NAT Chagas and the in-house assay. 

**Figure 2:**
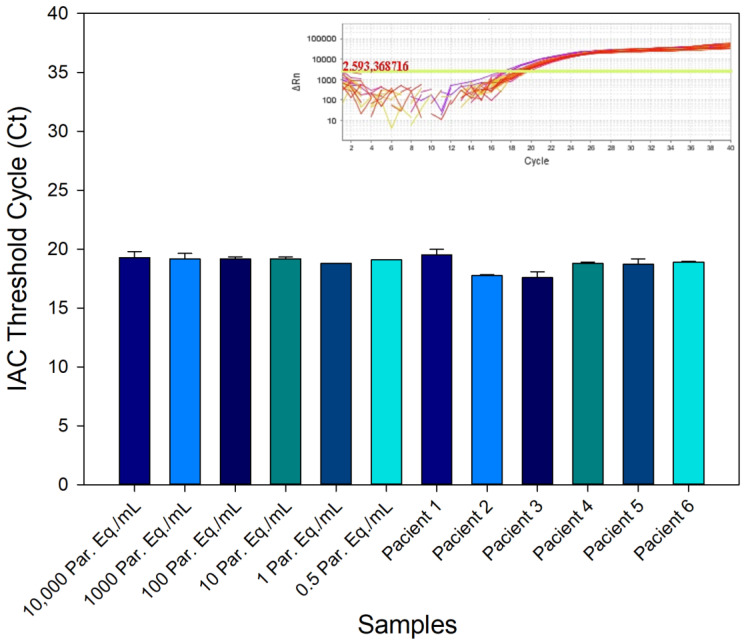
NAT Chagas internal amplification control (IAC) detection in samples with different *T. cruzi* concentrations. GEB samples containing different *T. cruzi* concentrations (from 10,000 to 0.5 Par. Eq./mL) and Chagas disease patient samples, which were negative (Patients 1, 2, and 3) or positive to PCR (Patients 4, 5, and 6), were spiked with the IAC synthetic DNA prior to DNA extraction. The IAC amplification using the NAT Chagas kit was evaluated. The inset shows the amplification plots to the IAC target.

The amplification reproducibility of IAC in samples containing variable amounts of *T. cruzi* was tested to evaluate the interference of *T. cruzi* load in the multiplex amplification of the IAC target. GEB samples spiked with different concentrations of *T. cruzi* (from 10,000 to 0.5 Par. Eq./mL) and samples from CD patients previously resulting in positive or negative PCR were compared in relation to the amplification of the IAC target. As can be seen in Figure 2, IAC showed amplifications corresponding to Ct values of 17.57 ± 0.51 to 19.50 ± 0.50. However, no statistical difference was observed between the Ct values, indicating the reproducibility of the IAC amplification between samples with high or low concentrations of parasites. 

**Figure 3:**
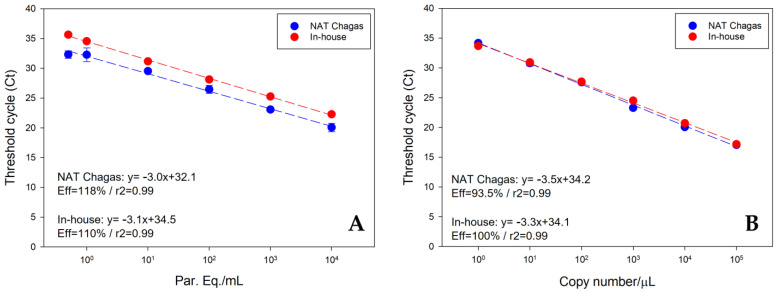
Comparative reportable range and linearity of qPCR assays with NAT Chagas and the in-house method. Multiplex TaqMan qPCR was carried out with DNA recovered from spiked GEB samples containing parasites (Y strain—TcII) in six concentrations spanning from 10^4^ to 0.5 Par. Eq./mL (**A**), and with *T. cruzi* synthetic satDNA mixed with human DNA in six concentrations spanning from 10^5^ to 1 copy number/µL (**B**). Each standard curve was tested in duplicate. The linear regression analyses are reported in the inset of each graph.

The dynamic extension of the in-house qPCR assay and the NAT Chagas were also compared. For the linearity assays, standard curves were constructed using DNA extracted from *T. cruzi* cells or from synthetic *T. cruzi* satDNA sequences. In both cases, to simulate the same matrix for the dilutions, containing the same characteristics and inhibitors, the parasite DNA or synthetic DNA were diluted in DNA extracted from a seronegative human blood containing a guanidine–EDTA solution (GEB). To carry out these experiments, the same standard curves were tested with the in-house qPCR and NAT Chagas. Figure 3A shows the linearity of the curve of DNA extracted from *T. cruzi* cells in GEB. It was possible to observe a dynamic range from 10^4^ to 0.5 parasite equivalents/mL in both assays, with PCR efficiencies of 110 and 118% for the in-house and NAT Chagas assays, respectively. Amplifications ranging from Ct 22.29 ± 0.10 to 35.65 ± 0.32 were observed for the in-house assay and Ct 20.07 ± 0.71 to 32.32 ± 0.63 for the NAT Chagas kit. At all points of the curves, it was possible to observe that the Cts for the reaction with NAT Chagas were about two units lower than those of the in-house assay, with the thresholds placed in the same position, showing an earlier amplification and fluorescence generation for the reaction with the kit.

Using the standard curve with the synthetic satDNA diluted in human DNA, a similar profile was observed, with a dynamic range from 10^5^ to 1 copy number/µL for both assays, with PCR efficiencies of 100% and 93.5% for the in-house and NAT Chagas assays, respectively. Amplifications ranging from Ct 17.19 ± 0.18 to 33.69 ± 0.11 were observed for the in-house assay and Ct 17.06 ± 0.22 to 34.18 ± 0.17 for the NAT Chagas kit.

**Figure 4:**
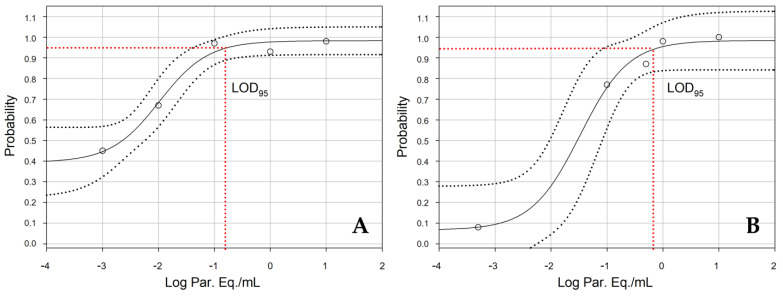
Probit regression (dose–response analysis) for the 95% limit of detection (LOD95) comparison between NAT Chagas and the in-house assay. (**A**). LOD95 assay for *T. cruzi* satDNA detection using NAT Chagas assay. (**B**). LOD95 assay for *T. cruzi* satDNA detection using the in-house assay. Each sample with different parasite loads was evaluated in 60 replicates for 5 consecutive days of experiments. The red dotted border corresponds to the estimates of LOD parameters with a 95% CI. The black lines represent the probit sigmoid dose-response curves, and the black dotted lines correspond to the 95% CI.

The 95% limit of detection (LOD95) of satDNA, expressed in Par. Eq./mL, was calculated as the lowest parasitic load that gives ≥95% of qPCR detectable results, according to the Clinical and Laboratory Standards Institute guidelines [35]. The LOD95 was estimated in DNA extracted from GEB samples spiked with known quantities of *T. cruzi* (Y strain—TcII) and analyzed with the NAT Chagas and the in-house assay. The recovered DNA from these spiked samples were amplified by qPCR for five consecutive days, for a total of 60 replicates for each parasite concentration. According to Probit regression analysis (Figure 4), the LOD95 established for the NAT Chagas assay was 0.16 Par. Eq./mL (Figure 4A), and, for the in-house assay, the LOD95 was 0.64 Par. Eq./mL (Figure 3B). Although the NAT Chagas kit showed a slightly lower LOD, no significant difference was found between the LOD values, showing that the sensitivity in detecting *T. cruzi* was similar for both methodologies.

Although the specificity of primers and probes used in both assays is described well in the literature [31,32,36], the specificity of the NAT Chagas kit was also evaluated in relation to the possibility of a crossed reaction with other protozoan species. For this purpose, 5 ng/µL of DNA from different pathogens was tested in the NAT Chagas kit, in parallel with positive *T. cruzi* controls (at 10 and 1 fg/µL). No amplification was observed for Leishmania (Viannia) braziliensis, Leishmania (Leishmania) amazonensis, Leishmania (Viannia) guyanensis, Leishmania (Leishmania) infantum, Plasmodium vivax, Plasmodium falciparum, Cyclospora cayetanensis, Eimeria acervuline, and Eimeria tenella, at the tested DNA concentration. However, for Toxoplasma gondii, a nonspecific amplification occurred. To further evaluate this interference of this cross-reaction, a serially diluted *T. gondii* tachyzoite DNA, from 10^6^ to 0.1 Par. Eq., was assayed in the NAT Chagas. No linearity in the amplification was observed, with Cts ranging from 34.51 ± 0.39 to 34.37 ± 0.26 relative to the highest and lowest concentrations tested, respectively. Since *T. gondii* is a protozoan parasite that is not usually detected in blood by PCR [40,41,42] and the clinical symptoms of Toxoplasmosis are easily differentiated from CD, this cross-reaction should not interfere with the performance of the NAT Chagas kit and other in-house qPCR assays that use the same primers and probe targeting *T. cruzi* satDNA.

### 3.2. Clinical Validation

During the previous stages of development, the NAT Chagas kit was tested with different blood samples from patients with chronic CD. However, the final clinical validation was performed with GEB samples from 50 patients from the northeast, southeast, south, and midwest regions of Brazil, consisting of 32 serological positive and 18 with negative serology for CD. Patients with chronic CD, who were 32 to 72 years old, and of whom 53.1% male and 46.9% female, with or without heart disease, from different states of Brazil, were probably infected during childhood by parasites from different DTUs (Supplementary Table S1). The same DNA samples, extracted from GEB as described in Materials and Methods, were tested in parallel with the NAT Chagas kit and the in-house assay. Of these patients, 19 had positive and 31 negative qPCR results in the in-house test. The results with the NAT Chagas kit showed 100% agreement with the in-house assay. Thus, considering the in-house qPCR as the gold standard for comparison, the NAT Chagas kit showed 100% sensitivity and 100% specificity in detecting *T. cruzi* DNA from GEB samples of chronic CD patients. In addition, when the qPCR results were compared to serology as the gold standard, 59.4% sensitivity and 100% specificity were observed (Table 2).

**Figure 5:**
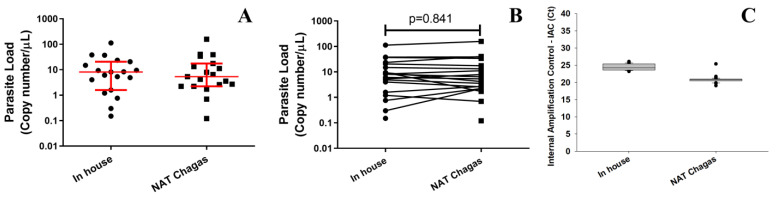
Comparison of the parasite load measures in chronic CD patients according to the use of the in-house qPCR and NAT Chagas assay followed by quantification of the IAC target in the GEB samples. (**A**). Parasite loads of the 19 chronic CD patients with positive results in the in-house (black dots) or NAT Chagas (black squares) assay; horizontal red lines and whiskers represent the medians and interquartile ranges of the values obtained. (**B**). Comparison of parasite loads in the in-house (black dots) or NAT Chagas (black squares) revealing no significant difference according to the paired statistical analysis performed between both qPCR assays (*p* = 0.841). (**C**). Box plot of the Ct values for the IAC in all tested GEB samples from chronic CD patients of the study. The dot outside the box represents the outlier.

The comparison of parasite load quantification in GEB samples of chronic CD patients, between NAT Chagas and the in-house assay, was carried out using the same synthetic DNA standard curve. The results were expressed in copy number/µL, as previously reported [39]. Figure 5A shows the overall parasite loads of the samples using both qPCR assays. In the in-house assay, the parasite loads ranged from 0.15 to 112.64 satDNA copies/µL with a median of 8.11 sat DNA copies/µL. In comparison, parasite loads ranging from 0.12 to 158.09 satDNA copies/µL were observed using the NAT Chagas kit, with a median of 5.35 satDNA copies/µL. When the parasite load values obtained by the two assays were compared (paired *t*-test), no significant difference was found (*p* = 0.841) (Figure 5B). Furthermore, to validate the reproducibility of the 50 DNA extractions and the quality of the samples, the Ct values for the IAC target were compared in each assay. For the in-house assay, the IAC Cts ranged from 23.20 to 26.07, with a mean value of 24.46. Using the NAT Chagas, the IAC Cts ranged from 19.07 to 21.74, with a mean value of 20.66, and there was one outlier sample (Ct 25.39) (Figure 5C).

**Figure 6:**
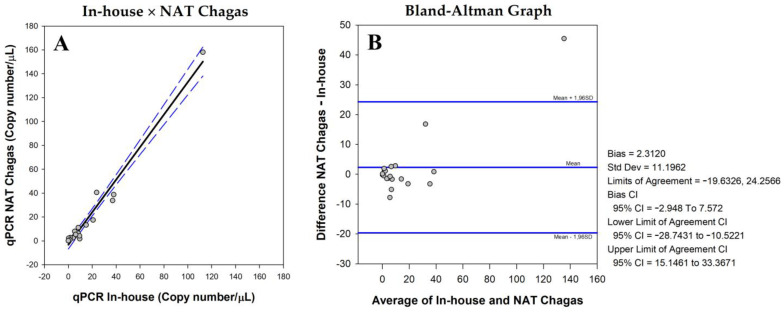
Agreement of the parasite load quantification between the in-house qPCR assay and NAT Chagas. (**A**). Parasite load plot (in-house × NAT Chagas). The black line represents the linear regression and the blue dashed lines represent the 95% CI. (**B**). Bland–Altman bias (difference) plot analysis for the degree of agreement between parasite load quantification using the in-house assay or NAT Chagas. On the right are the values of Bias, standard deviation, and limits of agreement. CI: Confidence interval. The central horizontal blue line represents the mean of the differences and the external horizontal blue lines represent the limits of agreement for the difference data. In (**A**,**B**), the dots represent the parasite load of each patient.

A Bland–Altman analysis [43] was used (Figure 6) to compare the parasite load quantification between both assays. It was possible to observe the similarity of the quantification results using the NAT Chagas kit and the in-house assay (Figure 6A). In the Bland–Altman plot (Figure 6B), the majority of samples were grouped around 0. Only 1 out of the 50 (2.0%) samples was positioned out of the limits of agreement (Figure 6B), demonstrating a very good concordance between both quantification assays. The estimated bias was only 2.31 satDNA copies/µL, meaning that the difference in the parasite load quantification between the assays was only 2.31 copies/uL.

When comparing the main commercial kits for the molecular diagnosis of CD, it is possible to observe that NAT Chagas has one of the lowest prices on the market (Table 3). In addition, it is the only kit registered by the Brazilian regulatory agency (ANVISA). It is noteworthy that kits produced in compliance with current regulations for products intended for in vitro diagnosis have a higher cost than those produced as inputs for research (research use only). Furthermore, most countries in South America adopt ANVISA as the reference regulatory agency; once the tests have been registered, the agency takes care of the final registration in the destination country.

## 4. Discussion

With the advent of CD molecular diagnosis, the use of PCR to detect minimal amounts of *T. cruzi* DNA directly in the blood of infected individuals has opened new possibilities for diagnosing chronic infections and evaluating etiologic treatment schemes [10,11,12,44,45,46,47]. Due to the high number of copies per parasite, kDNA [44] and satDNA [10] are widely used in the molecular diagnosis. However, there was no standardization regarding protocols, molecular targets and controls used in conventional and real-time PCR assays. Over the years, initiatives have emerged to establish methodological consensus and harmonization between the main tests used [35,36]. However, a few years ago, as only in-house PCR assays were available for routine use, the lack of standardized and validated commercial products hindered the reproducibility of clinical studies using PCR as a tool for the molecular diagnosis of CD and to monitor treatment failure.

Recently, new kits have been validated for the molecular diagnosis of CD [48,49,50]. Nevertheless, further and comparative evaluation of standardized qPCR kits is still needed in prospective blind-based studies. The NAT Chagas kit was developed in Brazil by the Instituto de Biologia Molecular do Paraná, in partnership with the Instituto Oswaldo Cruz/Fiocruz, based on the most reported qPCR assay in the literature as a product of an international consensus among specialists aiming to validate a qPCR method for the detection and quantification of *T. cruzi* DNA in blood [36]. The kit was designed to be easy to use and to simplify the analysis of the results, besides having positive and negative controls that aim to facilitate its application by any equipped laboratory to perform molecular diagnostic assays, even for other diseases. For this reason, we believe that the use of this kit will not be restricted to specialized centers; instead, it could be an important molecular tool for all laboratories that need to detect and quantify *T. cruzi* DNA in different types of samples. Here, we presented the comparative validation of the NAT Chagas kit with the in-house assay, using samples from chronic CD patients of different Brazilian states. Studies have shown that chronic patients in Brazil may be the ones with the lowest parasite burden, compared to CD patients from other endemic countries of Latin America, such as Argentina and Colombia [33,36]. Thus, the validation using blood samples from these patients indicates the robustness and high sensitivity of the NAT Chagas kit in relation to other different epidemiological scenarios.

By using a widely validated set of primers and probes [32], the high sensitivity and specificity of the NAT Chagas qPCR kit were expected. Indeed, it was observed that NAT Chagas successfully amplified *T. cruzi* DNA from all DTUs tested (TcI to TcVI), as previously reported for the in-house qPCR [31,32,36]. Even when using DNA extracted from the same number of *T. cruzi* cells (10^5^ Par. Eq./mL), we noted some variations in Ct values between parasites of different DTUs, probably due to differences in the number of satDNA copies between *T. cruzi* DTUs. It was previously observed that for the same amount of parasite DNA, TcI strains had higher Ct values than TcII, which would correspond to a lower number of satDNA copies of the former DTU [31,39,51,52]. In fact, recent findings have described that the size of the *T. cruzi* mitochondrial and nuclear genome and the content of chromosomes and multigene families vary in sequence and content among the parasite’s strains, even those classified into the same DTU or between different DTUs [53,54,55].

The NAT Chagas dynamic range and LOD95 were also compared to the in-house assay. The standard curves were very similar between them, especially when the synthetic DNA template was used. The same dynamic range was observed for both assays, which was similar to the one previously reported [32,36], thus showing the wide linearity of NAT Chagas to quantify parasite loads, in chronic and acute CD and in patients with disease reactivation. In addition, the NAT Chagas LOD95 was slightly lower than the in-house assay, but for both methods, the values fit between 0.1 and 1 Par. Eq./mL, as previously reported [32,36]. 

For the clinical validation, we selected a panel of samples from chronic CD patients born in different regions of Brazil, where they were probably infected during childhood. The genetic diversity of *T. cruzi* was also represented in these samples, with parasites from DTUs TcII, TcV, and TcVI and mixed infections by TcII + TcVI and TcIII + TcVI. However, as previously reported in Brazil, most patients are infected with TcII or TcVI [56,57,58]. Regardless of DTU and parasitic load, NAT Chagas presented similar results to the in-house test, showing high diagnostic accuracy of the kit. Compared to serology, NAT Chagas was able to detect *T. cruzi* DNA in 59.4% of patients with chronic CD. This is in line with reports in the literature [29,35], indicating that not all chronic patients have circulating parasites at the time of blood sample collection. This is expected, since chronic CD patients have very low and transient parasitemia, in contrast to the high level of anti-*T. cruzi* IgG [3].

So far, the NAT Chagas kit has only been registered by the National Health Surveillance Agency (ANVISA) of Brazil for the qualitative detection of *T. cruzi* DNA. Nevertheless, using the synthetic DNA that is supplied with the kit, a standard curve can be constructed for the absolute quantification of parasite load. Thus, we were also able to compare the in-house qPCR assay and the NAT Chagas for quantitative PCR. By using the Bland–Altman agreement method, we demonstrated very similar performance of the NAT Chagas kit compared to the in-house test concerning the absolute quantification of parasitic load. Only one of the fifty samples remained out of the agreement limits, showing a high concordance between the two assays.

Taken together, the validation results of NAT Chagas showed an excellent performance for its use in the molecular diagnosis of chronic CD patients, emerging as one of the main competitors of this pioneering generation of molecular kits for CD. This opens a perspective for the use of this kit in the diagnosis of patients under etiological treatment, newborns (infected by vertical transmission), immunosuppressed patients (transplanted and co-infected), and even acute (or recent chronic) patients, among others. As the NAT Chagas kit provides an exogenous internal control that can be used in different types of samples, the kit is also promising for the molecular diagnosis of oral outbreaks of CD. For this purpose, it must also be implemented for use with samples of açaí and other fruits after adapting the pre-PCR step (sample stabilization and DNA extraction). 

Among the main limitations of this study, we highlight the validation of the kit with a limited number of samples. However, this is justified by the complexity of obtaining samples from patients with chronic CD, even in Brazil. Another limitation is that all samples came from Brazil. Even so, as discussed above, patients were infected by *T. cruzi* from different DTUs, which softens the limited origin of the samples. We are currently gathering a larger pool of samples from chronic patients to expand the kit’s validation, as well as samples from acute, immunosuppressed, and infected individuals involved in oral outbreaks of CD in Brazil. 

Another important characteristic of NAT Chagas is that it is the only commercial kit for the molecular diagnosis of CD that has an option to be supplied with a sample stabilizing solution, composed of guanidine hydrochloride containing EDTA. Guanidine–EDTA is a lysis solution that allows the maintenance of blood samples at room temperature, thus facilitating the transport and conditioning of samples collected in the field to the laboratory where the molecular assays are to be carried out [45]. The use of this solution facilitates the recruitment of patients in remote regions with poor infrastructure, where generally CD is more prevalent, beyond reducing transport costs, which becomes strategic for this neglected disease.

## 5. Conclusions

Taking into account all the steps, including its production and distribution, as well as its cost and potential performance, the NAT Chagas kit emerges as an important alternative for the molecular diagnosis of CD not only in Brazil, which is the largest endemic country in the world, but also in other endemic countries in Latin America and countries where CD has become a public health problem due to the large number of immigrants from endemic areas in recent decades. Thus, this new generation of NAT kits may represent a milestone for the diagnosis of this neglected disease, improving the quality of life of thousands of people with CD around the world.

## Figures and Tables

**Table 1:** Sequences of *T. cruzi* satDNA and IAC synthetic controls.

Target	Sequence (5′-3′)	Length of Fragment (bp)	Sequence ID NCBI	Description
Nuclear Satellite DNA	AGTCGGCTGATCGTTTTCGAGCGGCTGCTACATCACACGTTGTGGTCTAAATTTTTGTTTCCAATTATGAATGGTGGGAGTCAGAGGCACTCTCTGTCACTATCTGTTTGCGTGTTCACACACTGGACACCAAACAACCCTGAATTATCCGCTGCTTGGAGGAATT	166	XM_805618.1	*Trypanosoma cruzi* strain CL Brener hypothetical protein Tc00.1047053508097.10 partial Mrna
Exogenous Internal Positive Control (IAC)	ACCGTCATGGAACAGCACGTACCGATTTATAAGATTGCTGGAGAAATGACTGGATTTGGAGCATCTGTTCTTGAAGGTGTTTTAGCTTTCGTCTTGGTTTATACTGTGTTCACGGCTAGCGATCCCAGACGTGGGCTACCTTTAGCAGTGGGACCTATATTTATAGGGTTTGTTGCGGGAG	181	NM_114612.3	*Arabidopsis thaliana* putative aquaporin TIP5-1 mRNA, complete cds

**Table 2:** Sensitivity and specificity of the NAT Chagas kit relative to the in-house qPCR assay or serology.

	In-House qPCR Assay × NAT Chagas		Serology × NAT Chagas	
	In-House +	In-House −	Serology +	Serology −
NAT Chagas +	19 (100%)	0 (0%)	19 (59.4%)	0 (0%)
NAT Chagas −	0 (0%)	31 (100%)	13 (40.6%)	18 (100%)
Total	19	31	32	18

**Table 3:** Leading kits for the molecular diagnosis of Chagas disease in the global market.

Product	Presentation (Reactions)	Price per Kit (USD)	Price per Reaction (USD)
A	50	556.00	11.12
B	30	346.00	11.53
C	100	450.00	4.50
D	50	750.00	15.00
E	100	1600.00	16.00
F	150	841.00	5.61
G	150	2526.00	16.84
NAT Chagas	96	703.00	7.55

## Data Availability

The original contributions presented in the study are included in the article/Supplementary Material; further inquiries can be directed to the corresponding author/s.

## Supplementary Materials

The following supporting information can be downloaded at: https://www.mdpi.com/article/10.3390/life13061236/s1, Table S1: Clinical validation data of the NAT Chagas kit.

## References

[1] World Health Organization Chagas Disease (American Trypanosomiasis). https://www.who.int/health-topics/chagas-disease.

[2] Alarcón de Noya B., Jackson Y., Pinazo Delgado M.J., Gascón J. (2020). Chagas Disease Epidemiology: From Latin America to the World. Chagas Disease.

[3] Rassi A., Rassi A., Marcondes de Rezende J. (2012). American Trypanosomiasis (Chagas Disease). Infect. Dis. Clin. N. Am..

[4] Antinori S., Corbellino M. (2018). Chagas Disease in Europe: A Long Way to Go. Eur. J. Intern. Med..

[5] Bern C., Messenger L.A., Whitman J.D., Maguire J.H. (2019). Chagas Disease in the United States: A Public Health Approach. Clin. Microbiol. Rev..

[6] Dias J.C.P., Ramos A.N., Gontijo E.D., Luquetti A., Shikanai-Yasuda M.A., Coura J.R., Torres R.M., Melo J.R.D.C., De Almeida E.A., De Oliveira Junior W. (2016). 2nd Brazilian Consensus on Chagas Disease, 2015. Rev. Soc. Bras. Med. Trop..

[7] Schijman A.G., Alonso-Padilla J., Longhi S.A., Picado A. (2021). Parasitological, Serological and Molecular Diagnosis of Acute and Chronic Chagas Disease: From Field to Laboratory. Mem. Inst. Oswaldo Cruz.

[8] Ordóñez D., Fernández-Soto P., Fernández-Martín A.M., Crego-Vicente B., Febrer-Sendra B., Diego J.G.B., Vicente B., López-Abán J., Belhassen-García M., Muro A. (2020). A *Trypanosoma cruzi* Genome Tandem Repetitive Satellite DNA Sequence as a Molecular Marker for a LAMP Assay for Diagnosing Chagas’ Disease. Dis. Markers.

[9] PAHO—Organización Panamericana de la Salud (2020). Síntesis de evidencia: Guía para el diagnóstico y el tratamiento de la enfermedad de Chagas. Rev. Panam. Salud Publica.

[10] Moser D.R., Kirchhoff L.V., Donelson J.E. (1989). Detection of *Trypanosoma cruzi* by DNA Amplification Using the Polymerase Chain Reaction. J. Clin. Microbiol..

[11] Avila H.A., Pereira J.B., Thiemann O., De Paiva E., Degrave W., Morel C.M., Simpson L. (1993). Detection of *Trypanosoma cruzi* in Blood Specimens of Chronic Chagasic Patients by Polymerase Chain Reaction Amplification of Kinetoplast Minicircle DNA: Comparison with Serology and Xenodiagnosis. J. Clin. Microbiol..

[12] Britto C., Cardoso M.A., Ravel C., Santoro A., Pereira J.B., Coura J.R., Morel C.M., Wincker P. (1995). *Trypanosoma cruzi*: Parasite Detection and Strain Discrimination in Chronic Chagasic Patients from Northeastern Brazil Using PCR Amplification of Kinetoplast DNA and Nonradioactive Hybridization. Exp. Parasitol..

[13] Britto C.C. (2009). Usefulness of PCR-Based Assays to Assess Drug Efficacy in Chagas Disease Chemotherapy: Value and Limitations. Mem. Inst. Oswaldo Cruz.

[14] Balouz V., Agüero F., Buscaglia C.A. (2017). Chagas Disease Diagnostic Applications: Present Knowledge and Future Steps. Adv. Parasitol..

[15] Schijman A.G., Altcheh J., Burgos J.M., Biancardi M., Bisio M., Levin M.J., Freilij H. (2003). Aetiological Treatment of Congenital Chagas’ Disease Diagnosed and Monitored by the Polymerase Chain Reaction. J. Antimicrob. Chem..

[16] Bua J., Volta B.J., Perrone A.E., Scollo K., Velázquez E.B., Ruiz A.M., De Rissio A.M., Cardoni R.L. (2013). How to Improve the Early Diagnosis of *Trypanosoma cruzi* Infection: Relationship between Validated Conventional Diagnosis and Quantitative DNA Amplification in Congenitally Infected Children. PLoS Negl. Trop. Dis..

[17] Cura C.I., Ramírez J.C., Rodríguez M., Lopez-Albízu C., Irazu L., Scollo K., Sosa-Estani S. (2017). Comparative Study and Analytical Verification of PCR Methods for the Diagnosis of Congenital Chagas Disease. J. Mol. Diag..

[18] Shikanai-Yasuda M.A., Carvalho N.B. (2012). Oral Transmission of Chagas Disease. Clin. Infec. Dis..

[19] de Noya B.A., González O.N. (2015). An Ecological Overview on the Factors That Drives to *Trypanosoma cruzi* Oral Transmission. Acta Trop..

[20] Ferreira R.T.B., Cabral M.L., Martins R.S., Araujo P.F., Da Silva S.A., Britto C., Branquinho M.R., Cardarelli-Leite P., Moreira O.C. (2018). Detection and Genotyping of *Trypanosoma cruzi* from Açai Products Commercialized in Rio de Janeiro and Pará, Brazil. Parasites Vectors.

[21] Finamore-Araujo P., Faier-Pereira A., Ramon do Nascimento Brito C., Gomes Peres E., Kazumy de Lima Yamaguchi K., Trotta Barroso Ferreira R., Moreira O.C. (2021). Validation of a novel multiplex real-time PCR assay for Trypanosoma cruzi detection and quantification in açai pulp. PLoS ONE.

[22] Diez M., Favaloro L., Bertolotti A., Burgos J.M., Vigliano C., Lastra M.P., Levin M.J., Arnedo A., Nagel C., Schijman A.G. (2007). Usefulness of PCR strategies for early diagnosis of Chagas’ disease reactivation and treatment follow-up in heart transplantation. Am. J. Transplant..

[23] Cura C.I., Lattes R., Nagel C., Gimenez M.J., Blanes M., Calabuig E., Iranzo A., Barcan L.A., Anders M., Schijman A.G. (2013). Early molecular diagnosis of acute Chagas disease after transplantation with organs from *Trypanosoma cruzi-*infected donors. Am. J. Transplant..

[24] Burgos J.M., Diez M., Vigliano C., Bisio M., Risso M., Duffy T., Cura C., Brusses B., Favaloro L., Leguizamon M.S. (2010). Molecular identification of *Trypanosoma cruzi* discrete typing units in end-stage chronic Chagas heart disease and reactivation after heart transplantation. Clin. Infect. Dis..

[25] de Freitas V.L., da Silva S.C., Sartori A.M., Bezerra R.C., Westphalen E.V., Molina T.D., Teixeira A.R., Ibrahim K.Y., Shikanai-Yasuda M.A. (2011). Real-time PCR in HIV/*Trypanosoma cruzi* coinfection with and without Chagas disease reactivation: Association with HIV viral load and CD4 level. PLoS Negl. Trop. Dis..

[26] Schijman A.G. (2018). Molecular Diagnosis of *Trypanosoma cruzi*. Acta Trop..

[27] CDC—Centers for Disease Control and Prevention American Tripanosomiasis. https://www.cdc.gov/dpdx/trypanosomiasisAmerican/index.html.

[28] Brasil P.E., De Castro L., Hasslocher-Moreno A.M., Sangenis L.H., Braga J.U. (2010). ELISA versus PCR for Diagnosis of Chronic Chagas Disease: Systematic Review and Meta-Analysis. BMC Infect. Dis..

[29] Ramírez J.D., Guhl F., Umezawa E.S., Morillo C.A., Rosas F., Marin-Neto J.A., Restrepo S. (2009). Evaluation of adult chronic Chagas’ heart disease diagnosis by molecular and serological methods. J. Clin. Microbiol..

[30] Piron M., Fisa R., Casamitjana N., López-Chejade P., Puig L., Vergés M., Gascón J., Prat J.G.i., Portús M., Sauleda S. (2007). Development of a Real-Time PCR Assay for *Trypanosoma cruzi* Detection in Blood Samples. Acta Trop..

[31] Duffy T., Bisio M., Altcheh J., Burgos J.M., Diez M., Levin M.J., Favaloro R.R., Freilij H., Schijman A.G. (2009). Accurate Real-Time PCR Strategy for Monitoring Bloodstream Parasitic Loads in Chagas Disease Patients. PLoS Negl. Trop. Dis..

[32] Duffy T., Cura C.I., Ramirez J.C., Abate T., Cayo N.M., Parrado R., Bello Z.D., Velazquez E., Muñoz-Calderon A., Juiz N.A. (2013). Analytical Performance of a Multiplex Real-Time PCR Assay Using TaqMan Probes for Quantification of *Trypanosoma cruzi* Satellite DNA in Blood Samples. PLoS Negl. Trop. Dis..

[33] Moreira O.C., Ramírez J.D., Velázquez E., Melo M.F.A.D., Lima-Ferreira C., Guhl F., Sosa-Estani S., Marin-Neto J.A., Morillo C.A., Britto C. (2013). Towards the Establishment of a Consensus Real-Time QPCR to Monitor *Trypanosoma cruzi* Parasitemia in Patients with Chronic Chagas Disease Cardiomyopathy: A Substudy from the BENEFIT Trial. Acta Trop..

[34] Qvarnstrom Y., Schijman A.G., Veron V., Aznar C., Steurer F., da Silva A.J. (2012). Sensitive and Specific Detection of *Trypanosoma cruzi* DNA in Clinical Specimens Using a Multi-Target Real-Time PCR Approach. PLoS Negl. Trop. Dis..

[35] Schijman A.G., Bisio M., Orellana L., Sued M., Duffy T., Mejia Jaramillo A.M., Cura C., Auter F., Veron V., Qvarnstrom Y. (2011). International Study to Evaluate PCR Methods for Detection of *Trypanosoma cruzi* DNA in Blood Samples from Chagas Disease Patients. PLoS Negl. Trop. Dis..

[36] Ramírez J.C., Cura C.I., Da Cruz Moreira O., Lages-Silva E., Juiz N., Velázquez E., Ramírez J.D., Alberti A., Pavia P., Flores-Chávez M.D. (2015). Analytical Validation of Quantitative Real-Time PCR Methods for Quantification of *Trypanosoma cruzi* DNA in Blood Samples from Chagas Disease Patients. J. Mol. Diag..

[37] Porrás A.I., Yadon Z.E., Altcheh J., Britto C., Chaves G.C., Flevaud L., Martins-Filho O.A., Ribeiro I., Schijman A.G., Shikanai-Yasuda M.A. (2015). Target Product Profile (TPP) for Chagas Disease Point-of-Care Diagnosis and Assessment of Response to Treatment. PLoS Negl. Trop. Dis..

[38] Alonso-Padillaid J., Abril M., de Noya B.A., Almeida I.C., Angheben A., Jorge T.A., Chatelain E., Esteva M., Gascón J., Grijalva M.J. (2020). Target Product Profile for a Test for the Early Assessment of Treatment Efficacy in Chagas Disease Patients: An Expert Consensus. PLoS Negl. Trop. Dis..

[39] Muñoz-Calderón A., Silva-Gomes N.L., Apodaca S., Alarcón de Noya B., Díaz-Bello Z., Souza L.R.Q., Costa A.D.T., Britto C., Moreira O.C., Schijman A.G. (2021). Toward the Establishment of a Single Standard Curve for Quantification of *Trypanosoma cruzi* Natural Populations Using a Synthetic Satellite Unit DNA Sequence. J. Mol. Diag..

[40] Santos F.F., Nascimento H., Muccioli C., Costa D.F., Rizzo L.V., Commodaro A.G., Belfort R. (2015). Detection of *Toxoplasma Gondii* DNA in Peripheral Blood and Aqueous Humor of Patients with Toxoplasmic Active Focal Necrotizing Retinochoroiditis Using Real-Time PCR. Arq. Bras. Oftalmol..

[41] Franzen C., Altfeld M., Hegener P., Hartmann P., Arendt G., Jablonowski H., Rockstroh J., Diehl V., Salzberger B., Fätkenheuer G. (1997). Limited Value of PCR for Detection of *Toxoplasma Gondii* in Blood from Human Immunodeficiency Virus-Infected Patients. J. Clin. Microbiol..

[42] Ajzenberg D., Lamaury I., Demar M., Vautrin C., Cabié A., Simon S., Nicolas M., Desbois-Nogard N., Boukhari R., Riahi H. (2016). Performance Testing of PCR Assay in Blood Samples for the Diagnosis of Toxoplasmic Encephalitis in AIDS Patients from the French Departments of America and Genetic Diversity of Toxoplasma gondii: A Prospective and Multicentric Study. PLoS Negl. Trop. Dis..

[43] Bland J.M., Altman D.G. (1986). Statistical methods for assessing agreement between two methods of clinical measurement. Lancet.

[44] Sturm N.R., Degrave W., Morel C., Simpson L. (1989). Sensitive Detection and Schizodeme Classification of *Trypanosoma cruzi* Cells by Amplification of Kinetoplast Minicircle DNA Sequences: Use in Diagnosis of Chagas’ Disease. Mol. Biochem. Parasitol..

[45] Avila H.A., Sigman D.S., Cohen L.M., Millikan R.C., Simpson L. (1991). Polymerase Chain Reaction Amplification of *Trypanosoma cruzi* Kinetoplast Minicircle DNA Isolated from Whole Blood Lysates: Diagnosis of Chronic Chagas’ Disease. Mol. Biochem. Parasitol..

[46] Britto C., Cardoso M.A., Wincker P., Morel C.M. (1993). A Simple Protocol for the Physical Cleavage of *Trypanosoma cruzi* Kinetoplast DNA Present in Blood Samples and Its Use in Polymerase Chain Reaction (PCR)-Based Diagnosis of Chronic Chagas Disease. Mem. Inst. Oswaldo Cruz.

[47] Wincker P., Britto C., Pereira J.B., Cardoso M.A., Oelemann W., Morel C.M. (1994). Use of a simplified polymerase chain reaction procedure to detect *Trypanosoma cruzi* in blood samples from chronic chagasic patients in a rural endemic area. Am. J. Trop. Med. Hyg..

[48] Abras A., Ballart C., Llovet T., Roig C., Gutiérrez C., Tebar S., Berenguer P., Pinazo M.J., Posada E., Gascón J. (2018). Introducing Automation to the Molecular Diagnosis of *Trypanosoma cruzi* Infection: A Comparative Study of Sample Treatments, DNA Extraction Methods and Real-Time PCR Assays. PLoS ONE.

[49] Longoni S.S., Pomari E., Antonelli A., Formenti F., Silva R., Tais S., Scarso S., Rossolini G.M., Angheben A., Perandin F. (2020). Performance Evaluation of a Commercial Real-Time PCR Assay and of an in-House Real-Time PCR for *Trypanosoma cruzi* DNA Detection in a Tropical Medicine Reference Center, Northern Italy. Microorganisms.

[50] Benatar A.F., Danesi E., Besuschio S.A., Bortolotti S., Cafferata M.L., Ramirez J.C., Albizu C.L., Scollo K., Baleani M., Lara L. (2021). Prospective Multicenter Evaluation of Real Time PCR Kit Prototype for Early Diagnosis of Congenital Chagas Disease. eBioMedicine.

[51] Vargas N., Pedroso A., Zingales B. (2004). Chromosomal Polymorphism, Gene Synteny and Genome Size in *T. cruzi* I and *T. cruzi* II Groups. Mol. Biochem. Parasitol..

[52] Souza R.T., Lima F.M., Barros R.M., Cortez D.R., Santos M.F., Cordero E.M., Ruiz J.C., Goldenberg S., Teixeira M.M.G., da Silveira J.F. (2011). Genome Size, Karyotype Polymorphism and Chromosomal Evolution in *Trypanosoma cruzi*. PLoS ONE.

[53] Reis-Cunha J.L., Coqueiro-Dos-Santos A., Pimenta-Carvalho S.A., Marques L.P., Rodrigues-Luiz G.F., Baptista R.P., Almeida L.V., Honorato N.R.M., Lobo F.P., Fraga V.G. (2022). Accessing the Variability of Multicopy Genes in Complex Genomes using Unassembled Next-Generation Sequencing Reads: The Case of *Trypanosoma cruzi* Multigene Families. mBio.

[54] Wang W., Peng D., Baptista R.P., Li Y., Kissinger J.C., Tarleton R.L. (2021). Strain-specific genome evolution in *Trypanosoma cruzi*, the agent of Chagas disease. PLoS Pathog..

[55] Reis-Cunha J.L., Rodrigues-Luiz G.F., Valdivia H.O., Baptista R.P., Mendes T.A., de Morais G.L., Guedes R., Macedo A.M., Bern C., Gilman R.H. (2015). Chromosomal copy number variation reveals differential levels of genomic plasticity in distinct *Trypanosoma cruzi* strains. BMC Genom..

[56] Brenière S.F., Waleckx E., Barnabé C. (2016). Over Six Thousand *Trypanosoma cruzi* Strains Classified into Discrete Typing Units (DTUs): Attempt at an Inventory. PLoS Negl. Trop. Dis..

[57] Zingales B., Miles M.A., Campbell D.A., Tibayrenc M., Macedo A.M., Teixeira M.M.G., Schijman A.G., Llewellyn M.S., Lages-Silva E., Machado C.R. (2012). The Revised *Trypanosoma cruzi* Subspecific Nomenclature: Rationale, Epidemiological Relevance and Research Applications. Infect. Genet. Evol..

[58] Zingales B. (2018). *Trypanosoma cruzi* Genetic Diversity: Something New for Something Known about Chagas Disease Manifestations, Serodiagnosis and Drug Sensitivity. Acta Trop..

