# Supplementary material for: Validation of the NAT Chagas IVD Kit for the Detection and Quantification of Trypanosoma cruzi in Blood Samples of Patients with Chagas Disease
Source: Life (Basel). 2023 May 24;13(6):1236. doi: 10.3390/life13061236 (PMC10300704; doi:10.3390/life13061236)
Supplement: Supplementary file 1 [file life-13-01236-s001.zip › life-2323140-supplementary.pdf]

Table S1. Clinical validation data of the NAT Chagas kit. Fifty DNA samples were extracted from guanidine-EDTA blood of patients with Chagas disease and volunteers from non-endemic areas. All samples were analyzed for the NAT Chagas kit in parallel with the in-house assay. The characteristics of the samples and results for the qPCR using the NAT Chagas kit and the in-house assay are shown.

Characteristics of the samples							In house assay						NAT Chagas					
**Sample #**	**Serology Results**	**Age**	**Gender**	**Clinical Manifestation**	**DTU**	**Brazilian State**	**CT Mean IAC**	**SD IAC**	**CT Mean satDNA**	**SD satDNA**	**Parasite load (copy number/µL)**	**SD Parasite load**	**CT Mean IAC**	**SD IAC**	**CT Mean satDNA**	**SD satDNA**	**Parasite load (copy number/µL)**	**SD Parasite load**
**1**	Positive	61	female	indeterminate	II	RS	25.22	0.12	30.77	0.43	8.31	2.44	20.71	0.19	30.6	0.38	11.12	2.83
**2**	Positive	48	female	heart disease	II	CE	25.61	0.16	34.1	1.04	8.11	4.7	20.94	0.07	31.61	0.75	6.44	3.14
**3**	Positive	53	female	indeterminate	II	PE	23.58	0.09	29.32	0.42	20.70	5.70	20.71	0.12	29.78	0.70	17.48	6.29
**4**	Positive	61	female	indeterminate	-	MG	26.07	0.23	ND	ND	ND	ND	21.34	0.22	ND	ND	ND	ND
**5**	Positive	48	female	indeterminate	-	CE	23.91	0.02	ND	ND	ND	ND	20.76	0.09	ND	ND	ND	ND
**6**	Positive	42	male	indeterminate	II	MG	23.68	0.07	34.5	0.09	0.76	0.05	21.19	0.16	34.02	2.28	2.22	2.38
**7**	Positive	46	male	indeterminate	II/V/VI	RJ	23.46	0.08	31.6	0.7	4.91	2.56	20.6	0.17	32.5	0.95	3.57	2
**8**	Positive	42	male	indeterminate	II/V/VI	AL	23.49	0.05	29.91	0.19	14.89	1.98	20.63	0.18	30.19	0.33	13.3	2.51
**9**	Positive	41	male	indeterminate	-	CE	23.59	0.09	ND	ND	ND	ND	20.79	0.25	ND	ND	ND	ND
**10**	Positive	44	male	heart disease	-	CE	23.74	0.15	ND	ND	ND	ND	21	0.39	ND	ND	ND	ND
**11**	Positive	72	female	indeterminate	-	MG	23.51	0.04	ND	ND	ND	ND	19.94	0.14	ND	ND	ND	ND
**12**	Positive	54	male	heart disease	II/VI	PE	25.65	0.03	33.71	0.68	9.53	4.04	20.85	0.13	33.73	0.52	1.72	0.53
**13**	Positive	39	male	indeterminate	II/VI	AL	23.59	0.03	31.48	0.72	5.33	2.61	20.82	0.08	31.17	0.91	7.89	3.59
**14**	Positive	56	female	indeterminate	II+VI	RJ	24.25	0.17	29.09	0.24	23.71	3.74	21.59	0.06	28.31	0.3	40.52	7.32
**15**	Positive	49	female	indeterminate	II+VI	AL	23.79	0.08	28.61	0.18	37.06	4.48	20.91	0.08	28.7	0.52	33.82	9.93
**16**	Positive	57	male	heart disease	-	CE	25.67	0.05	ND	ND	ND	ND	20.97	0.02	ND	ND	ND	ND
**17**	Positive	36	female	heart disease	II+VI	CE	25.38	0.25	33.9	1.06	9.32	5.76	21.03	0.09	32.32	0.9	4.19	2.14
**18**	Positive	68	female	heart disease	III+VI	BA	25.64	0.19	35.04	0.67	4.04	1.7	20.74	0.24	33.09	0.24	2.6	1.16
**19**	Positive	57	female	heart disease	V	BA	25.85	0.04	36.39	0.07	1.6	0.07	20.76	0.28	33.62	2.21	2.7	2
**20**	Positive	53	male	indeterminate	V	BA	23.59	0.15	28.39	0.10	37.95	2.46	20.66	0.10	28.44	0.60	38.79	13.41
**21**	Positive	66	male	indeterminate	VI	GO	23.48	0.02	36.11	1.23	0.3	0.23	21.19	0.16	34.02	2.28	2.22	2.38
**22**	Positive	61	male	indeterminate	-	PB	24.55	0.08	ND	ND	ND	ND	21.74	0.11	ND	ND	ND	ND
**23**	Positive	68	female	indeterminate	VI	BA	25.42	0.06	36.54	1.61	0.15	0.14	20.89	0.22	ND	ND	ND	ND

**24**	Positive	38	male	heart disease	-	CE	25.22	0.12	ND	ND	ND	ND	20.75	0.08	ND	ND	ND	ND
**25**	Positive	32	male	heart disease	VI	PB	25.93	0.18	34.12	0.97	1.20	0.65	20.89	0.10	35.44	1.95	0.70	0.51
**26**	Positive	56	female	indeterminate	-	BA	23.69	0.03	ND	ND	ND	ND	21.24	0.05	ND	ND	ND	ND
**27**	Positive	45	male	indeterminate	-	MG	26.01	0.18	ND	ND	ND	ND	21.25	0.28	ND	ND	ND	ND
**28**	Positive	67	female	heart disease	-	SE	25.23	0.06	ND	ND	ND	ND	20.88	0.19	ND	ND	ND	ND
**29**	Positive	66	male	heart disease	VI	BA	23.44	0.12	27.09	0.11	112.64	8.52	20.78	0.28	26.4	0.09	158.09	9.22
**30**	Positive	67	male	heart disease	-	MG	23.77	0.12	ND	ND	ND	ND	21.22	0.12	ND	ND	ND	ND
**31**	Positive	60	female	heart disease	VI	AL	25.43	0.12	34.66	1.26	6.09	4.73	20.86	0.05	31.89	0.67	5.35	2.33
**32**	Positive	58	male	indeterminate	-	PB	25.13	0.15	ND	ND	ND	ND	20.52	0.28	38.1	2.17	0.12	0.13
**33**	Negative	30	female	-	-	RJ	24.48	0.19	ND	ND	ND	ND	19.85	0.27	ND	ND	ND	ND
**34**	Negative	22	female	-	-	RJ	25.63	0.12	ND	ND	ND	ND	20.9	0.3	ND	ND	ND	ND
**35**	Negative	25	male	-	-	RJ	25.49	0.8	ND	ND	ND	ND	21.23	0.07	ND	ND	ND	ND
**36**	Negative	23	female	-	-	RJ	23.83	0.55	ND	ND	ND	ND	19.07	0.23	ND	ND	ND	ND
**37**	Negative	28	male	-	-	RJ	24.29	0.06	ND	ND	ND	ND	19.77	0.1	ND	ND	ND	ND
**38**	Negative	21	female	-	-	RJ	23.99	0.2	ND	ND	ND	ND	19.89	0.06	ND	ND	ND	ND
**39**	Negative	32	female	-	-	RJ	24.33	0.15	ND	ND	ND	ND	20.01	0.06	ND	ND	ND	ND
**40**	Negative	22	female	-	-	RJ	25.39	0.04	ND	ND	ND	ND	25.39	0.04	ND	ND	ND	ND
**41**	Negative	41	male	-	-	RJ	25.1	0.23	ND	ND	ND	ND	20.5	0.15	ND	ND	ND	ND
**42**	Negative	28	male	-	-	RJ	23.2	0.38	ND	ND	ND	ND	19.77	0.1	ND	ND	ND	ND
**43**	Negative	25	female	-	-	RJ	23.92	0.33	ND	ND	ND	ND	20.52	0.2	ND	ND	ND	ND
**44**	Negative	33	female	-	-	RJ	24.38	0.07	ND	ND	ND	ND	20.79	0.11	ND	ND	ND	ND
**45**	Negative	26	female	-	-	RJ	23.81	0.04	ND	ND	ND	ND	19.99	0.1	ND	ND	ND	ND
**46**	Negative	35	female	-	-	RJ	23.44	0.23	ND	ND	ND	ND	19.9	0.15	ND	ND	ND	ND
**47**	Negative	31	male	-	-	RJ	23.51	0.04	ND	ND	ND	ND	19.94	0.14	ND	ND	ND	ND
**48**	Negative	30	female	-	-	RJ	24.53	0.05	ND	ND	ND	ND	21.24	0.09	ND	ND	ND	ND
**49**	Negative	27	female	-	-	RJ	23.2	0.26	ND	ND	ND	ND	19.75	0.06	ND	ND	ND	ND
**50**	Negative	28	female	-	-	RJ	23.92	0.07	ND	ND	ND	ND	20.45	0.32	ND	ND	ND	ND

DTU: Discrete typing unit. SD: Standard deviation. ND: No Detectable.
